# 731 A Distressing Trend: Increased Prevalence of Self-Immolation Burns in a Single Burn Center

**DOI:** 10.1093/jbcr/irae036.274

**Published:** 2024-04-17

**Authors:** Jamie L Hollowell, Eli Maxwell, Booker King, Felicia Williams

**Affiliations:** University of North Carolina, Chapel Hill, NC; UNC Chapel Hill Jaycee Burn Center, Chapel Hill, NC; UNC Chapel Hill. NC, Chapel Hill, NC; The University of North Carolina at Chapel Hill, Chapel Hill, NC; University of North Carolina, Chapel Hill, NC; UNC Chapel Hill Jaycee Burn Center, Chapel Hill, NC; UNC Chapel Hill. NC, Chapel Hill, NC; The University of North Carolina at Chapel Hill, Chapel Hill, NC; University of North Carolina, Chapel Hill, NC; UNC Chapel Hill Jaycee Burn Center, Chapel Hill, NC; UNC Chapel Hill. NC, Chapel Hill, NC; The University of North Carolina at Chapel Hill, Chapel Hill, NC; University of North Carolina, Chapel Hill, NC; UNC Chapel Hill Jaycee Burn Center, Chapel Hill, NC; UNC Chapel Hill. NC, Chapel Hill, NC; The University of North Carolina at Chapel Hill, Chapel Hill, NC

## Abstract

**Introduction:**

Attempting suicide via self-immolation is one of the more traumatic injuries leading to burn center admission. Patients with acute or acute-on-chronic suicidality are now faced with coping with a new trauma, and often were poorly resourced prior to burn center admission. These patients require a multidisciplinary approach to optimize their healing while addressing their mental health. We aimed to describe the increase in prevalence of self-immolation burns in our burn center.

**Methods:**

Patients were identified using the Institutional Burn Center registry and linked to the clinical and administrative data. All patients admitted to the BICU between January 1, 2018, to Sept 30, 2023, were included. Demographics, length of stay (LOS), co-morbid conditions, and mortality were evaluated.

**Results:**

There were a total of 45 patients over the 5-year, 9 month period. There were six self-immolations each year for 2018-2020. There were eight self-immolation admissions for 2021, nine for 2022, and ten for 2023, as of September 30, 2023. The patients were majority male, with 31 males and 14 females; average age was 35.4, (12-69); average TBSA was 21% (0 to 95%), most common etiology was flame with accelerant.

**Conclusions:**

There continues to be a mismatch between the need for and access to mental health providers and resources in the community at large, leading to an yearly increase in the frequency of self-immolation admissions in our burn unit. These patients present unique challenges that require multidisciplinary coordination, with many patients still lacking appropriate disposition at discharge, taking up valuable burn center beds. Self-immolation as a form of suicide adds a new layer of emotional trauma for the patient, and often for the staff, which can make their recovery and rehabilitation that much more difficult. The ongoing mental health crisis in our country will likely see a further increase in these tragic injuries and the burn community needs to develop additional prevention and treatment strategies for this population.

**Applicability of Research to Practice:**

This study reinforces the need for additional mental health resources with enhanced access for the community at large. The increased admission rate demonstrates an ongoing need for psychiatric care in the at-risk burn trauma population.

